# Use of Saliva Biomarkers to Monitor Efficacy of Vitamin C in Exercise-Induced Oxidative Stress

**DOI:** 10.3390/antiox6010005

**Published:** 2017-01-12

**Authors:** Levi W. Evans, Stanley T. Omaye

**Affiliations:** Nutrition Program, Agriculture, Nutrition and Veterinary Science Department, University of Nevada, Reno, NV 89557, USA; levi659@gmail.com

**Keywords:** saliva, oxidative stress, vitamin C, ascorbic acid, malondialdehyde, exercise-induced oxidative stress

## Abstract

Saliva is easily obtainable for medical research and requires little effort or training for collection. Because saliva contains a variety of biological compounds, including vitamin C, malondialdehyde, amylase, and proteomes, it has been successfully used as a biospecimen for the reflection of health status. A popular topic of discussion in medical research is the potential association between oxidative stress and negative outcomes. Systemic biomarkers that represent oxidative stress can be found in saliva. It is unclear, however, if saliva is an accurate biospecimen as is blood and/or plasma. Exercise can induce oxidative stress, resulting in a trend of antioxidant supplementation to combat its assumed detriments. Vitamin C is a popular antioxidant supplement in the realm of sports and exercise. One potential avenue for evaluating exercise induced oxidative stress is through assessment of biomarkers like vitamin C and malondialdehyde in saliva. At present, limited research has been done in this area. The current state of research involving exercise-induced oxidative stress, salivary biomarkers, and vitamin C supplementation is reviewed in this article.

## 1. Introduction

**Figure 1:**
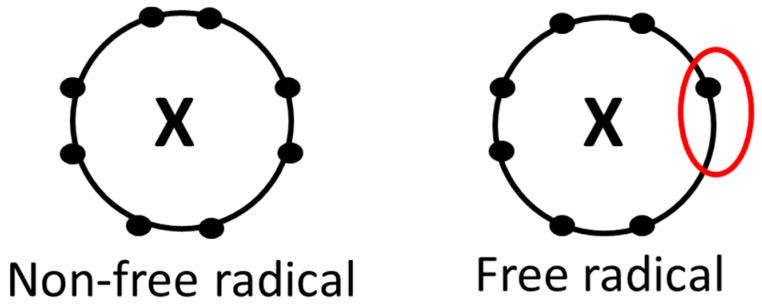
An example of a non-free radical (all electrons within orbital are paired) and a free radical (at least one electron within orbital is unpaired). The superoxide radical has an unpaired electron within its orbital; the unpaired electron creates a reactive compound capable of producing oxidative damage [2,3].

Free radicals and their potentially destructive nature were recognized in a laboratory as early as 1900 [1]. A free radical, for the purpose of this review, is defined as a compound that has one or more unpaired electrons in its orbital and is capable of finite existence (Figure 1) and includes superoxide [2,3]. Free radicals have extremely high chemical reactivity, which can in turn cause damage to cells. Reference 1 researchers investigating carbons and hydrocarbons acknowledged that oxygen derivatives were causing reactions within their experiments [1]. “Oxidation” was termed as the environment’s oxygen as it bound with chemical constituents. Since this century-old occurrence, studies have continued to examine oxidation, its properties, and how it can affect a multitude of health-related outcomes. The adverse effects of oxidation and free radicals were hypothesized by the mid-1900s and further examined by Gerschman et al. [4], who compared and identified the dangers of oxidation and radiation. Their findings led to future research more closely examining free radicals and consequences associated with their presence. It should be noted that the generation of free radicals is not always negative. A number of reactions essential to life, such as the generation of phagocytic cells to kill invading pathogens, along with those related to intercellular and intracellular signaling, are important beneficial effects.

Free radical species include reactive oxygen species (ROS), reactive nitrogen species (RNS), and a variety of compounds that can become reactive from surrounding free radicals; such compounds can include carbons, carbonyls, lipids, proteins, sulfur, halogen and nucleotides [5,6,7,8,9,10,11,12,13]. It can be argued that with time, individual organisms are exposed to more and more oxidizing elements resulting in more free radicals. These oxidizing elements are found in everyday life. Theoretically speaking, this would cause an accumulation of free radical damage needing to be repaired in order to avoid the correlated dangers of such instances. Antioxidants can reduce free radicals to less-harmful compounds at the expense of such antioxidants, which in turn become oxidized. One factor recognized as causing increased oxidation is exercise, known as exercise-induced oxidative stress. Davies was one of the first who found that exercise can induce oxidative stress as exhaustive exercise increased free radical production [14].

Biomarkers of oxidative stress have been a major source of debate related to monitoring oxidative stress and the possible resulting damage. Biomarkers of oxidative stress have been measured in plasma, whole blood, urine, respired gases, muscle, and other skeletal tissues. One possible biospecimen that is still requiring investigation is saliva, as it has the potential to be utilized for measuring a variety of biomarkers in relation to antioxidants and oxidative stress. Saliva is an attractive biospecimen for a number of reasons including the ease of its collection and the high-amount the human body is capable of producing for examination. Because indices for oxidative stress increase in exercise, dietary supplements are often used to counter such negative effects as soreness and fatigue. Furthermore, dietary supplements are often used to enhance exercise performance. Vitamin C is an antioxidant that has been used as a dietary supplement to mitigate exercise-induced oxidative stress, the negative results of unchecked free radicals, and the possible alterations in exercise-induced adaptation that might come of the vitamin’s consumption. This review paper will address these areas with the inclusion of the most recent research.

## 2. Oxidative Stress and Free Radicals

### 2.1. Basic Concepts

Reduction and oxidation reactions, or redox reactions, involve the process of electron transport between molecular orbitals. When a compound is reduced, it gains an electron in its orbital for stabilization; alternatively, when a compound is oxidized, it loses an electron from its orbital. Oxidization usually leads to an unpaired electron, turning it into a free radical or a compound known as a reactive species. Redox reactions are coupled in systems such as biological organisms; normally, these are balanced and otherwise healthy reactions unless some sort of “stress” should cause an imbalance between reduction and oxidation. The imbalance is usually skewed towards oxidation and is defined as oxidative stress; that is, a buildup of reactive species/free radicals (such as reactive oxygen species or reactive nitrogen species) which are not being reduced at an equal pace by antioxidants and leading to potential damage [15].

It should be recognized that oxygen is one of the more toxic chemicals in oxidative stress when not reduced. Oxygen is, however, a necessity for a wide range of organisms, being the final acceptor in electron transportation during ATP synthesis. Because of oxygen’s molecular structure, it is very capable of free radical formation and contributes highly to reactive oxygen species as a whole. This is interesting, given not only its biological necessity, but also given it is the most abundant chemical in the Earth’s atmosphere at 21% [16,17]. Exposure to high concentrations of oxygen or to anything leading to toxic concentrations of oxygen that cannot be reduced is known to be detrimental to organisms such as plants, animals, and bacteria [18,19,20]. To combat oxygen toxicity and its role in free radicals formation, such organisms require antioxidant defenses from both endogenous and exogenous sources. Humans are especially susceptible to high concentrations of oxygen exposure in the lungs because of their physiological function [21], but other tissues are capable of high concentrations as well. Oxygen at lower concentrations (e.g., hypoxia) can also produce biomarkers of oxidative stress [2]; this suggests oxygen’s balance is an important factor in oxidative stress.

The simplest free radical is atomic hydrogen. Free radicals can be highly reactive to other components and compounds in that they search for electrons to pair with their unpaired electrons. Such electrons often come from nucleotides, lipids, proteins, and/or other molecules throughout the surrounding system that are paramagnetic. This process, in turn, oxidizes these substances, potentially resulting in an array of problems such as mutation and dysfunction. The superoxide radical is one of the more common free radicals; it can either oxidize substances or create other free radicals [2]. Other known free radicals are hydroperoxyl, hydroxyl, peroxyl, alkoxyl, carbonate, carbon dioxide, and O_2_ [22]. Some compounds are considered non-radicals but have still been associated with oxidative stress, such as hydrogen peroxide, peroxynitrite, peroxynitrous acid, nitrosoperoxycarbonate, hypochlorous acid, hypobromous acid, ozone, and singlet O_2_. Nitrogen, chlorine, bromine, and sulfur have also been shown to produce their own versions of reactive species [23]. Free radicals can be formed under a variety of different circumstances including cell/tissue injury [24] and exercise.

### 2.2. Health, Disease, and Disorders Associated with Oxidative Stress

Based on the existing research on free radicals and oxidative stress and their potential association with negative outcomes, it is easy to misinterpret the available information and conclude that these are unhealthy and (at times) detrimental compounds. Possible cell death can occur via oxidative stress and free radical exposure. The list of clinical conditions associated with oxidative stress and free radicals is exhaustive; however, whether they are causative for these conditions or simply byproducts is not yet established. Some examples of research involving health conditions and their associations with free radicals are described below:

Porphyria is a condition in humans where heme’s biosynthesis is abnormal in such a way that leads to skin and tissue damage in severe cases, and often, a buildup of free radicals [25,26]. Atherosclerosis and cardiovascular disease are two of the leading causes of death worldwide [27,28]. Inflammatory and oxidative biomarkers (C-reactive protein and Myeloperoxidase) may accompany these conditions (and therefore are associated) [29]. Diabetes (among the 10 leading causes of death in the USA [30]) has been induced through introduction of oxidizing-toxins in laboratory conditions [31,32]. Furthermore, biomarkers of oxidative stress are shown to be increased with diabetes [33,34]. Reactive species and compounds that can generate reactive species have been shown to generate more rapidly during times of carcinogenesis and are increased in tumors [35,36]; cancer is another condition among the leading causes of death in the USA [30]. Research has established free radical production plays a role in aging. Researchers are working to establish the potential relationship between varying free radical and antioxidant concentrations as they relate to cell life and longevity [37,38,39,40].

Oxidation and free radical production cannot be avoided entirely, as organisms are both exposed to them from exogenous sources and through endogenous production. Air pollution, physical activity, high-altitude/hypoxia, inactivity, food, obesity, and UV-light exposure can cause oxidative damage. Furthermore, the mitochondria and phagocytes within the body can cause the production of free radicals [41]. It has been established that in certain biochemical/cellular situations, free radicals and reactive species are necessary for development and function such as in cell growth/proliferation, cell signaling, gene activation, and gene messaging just to name a few [42,43]. Production of reducing agents such as superoxide dismutase (SOD), catalase, and glutathione peroxidase (GPX) occurs endogenously to maintain a balanced redox system [2,44,45]. Exogenous sources of reducing agents, such as vitamin C and vitamin E, also have roles in this redox balance. These reducing agents are better known as antioxidants.

Antioxidants, for the sake of this review, are compounds capable of reducing oxidized compounds, free radicals, reactive species, and oxidative stress. Briefly mentioned earlier, antioxidants can be produced endogenously (e.g., SOD, catalase, and GPX) as well as sourced exogenously (e.g., vitamin C and vitamin E). The antioxidant used to reduce or alleviate the offending agent or oxidative stress depends on: the offending agent (e.g., free radical or reactive species); how it is generated (e.g., exercise); where it is generated (e.g., water-based tissue or lipid-based tissue, intracellularly or extracellularly); and the target of damage (e.g., plasma lipids or plasma proteins) [2]. Furthermore, antioxidants can defend differently against free radicals and/or reactive species; antioxidants SOD, GPX, and catalase act as enzymes and catalytically remove the offender while vitamin C, vitamin E, and reduced glutathione donate electrons to neutralize the offender. Antioxidants that donate electrons in order to reduce oxidative stress, do so at their own expense in that they become oxidized albeit a less harmful/oxidizing agent. Certain antioxidants are more capable of reducing oxidative stress more readily; endogenous sources, such as GPX, are the more capable compounds regarding this while vitamin C and vitamin E are less-so. Still, vitamin C and its related compounds (e.g., dehydroascorbic acid) are more capable than vitamin E and, at times, some endogenous antioxidants at reducing oxidative stress [2].

### 2.3. The Relationship between Inflammation and Oxidative Stress

Pathogens, trauma, and infections are common offenders which can cause the body to become inflamed. Inflammation is the mechanism used to restrict any sort of possible spreading, remove the debris from the damage, and repair the damaged area caused by the offender [46]. The first response to an offending agent such as a pathogen is to quickly mobilize defending leukocytes (such as monocytes and neutrophils) by vasodilation via cytokines. Cytokines such as interleukins and tumor necrosis factors will formulate to signal surrounding sensors to begin the necessary mechanisms for repair and restoration [47]; these are common inflammatory biomarkers. The next response is for leukocytes and monocytes to engulf debris that might result from the damage. Neutrophils will also catalyze a reaction known as a respiratory burst in order to neutralize the offender. Superoxide radical, hydrogen peroxide, and hypochlorite are the neutralizing compounds produced by the respiratory burst [48,49]; these are also reactive and non-reactive species of oxidative stress. The final response of inflammation includes cleanup by monocytes (and eventually macrophages) via phagocytosis [50] and then repair. The known signs of inflammation include redness, swelling, and pain. Inflammation and the aforementioned process are normal in acute situations, much like redox reactions.

It is when chronic inflammation and its mechanism arise, so do dangerous problems. The acute inflammatory response differs from the chronic inflammatory response. Cytokines such as interleukins and tumor necrosis factors tend to be overproduced. This can result in systemic inflammation which may be due to an over-abundance of adipose tissue [51]. Metabolic functions will then be disturbed. A variety of diseases have been linked to inflammation from this situation including obesity and diabetes [52], cardiovascular disease [53], and cancer [54]. 

Both acute inflammation and chronic inflammation will affect, and be affected by, redox components. It has been suggested reactive species (especially 8-oxo-2′-deoxyguanosine (8-oxo-dG), an oxidized derivative of deoxyguanosine and product of DNA oxidation) damaging DNA might be the main culprit in developing metabolic diseases such as cancer [55]. Cancer tumors exhibit an inflammatory response via increases in cytokines such as nuclear factor kappa beta (NF-κB) and tumor necrosis factor alpha (TNFα) [2]; as do situations in cardiovascular disease, diabetes, and hypertension with reactive species being possible initiators [56]. Oxidative stress might even be a secondary process to chronic inflammation [57]. Production of reactive species via inflammatory response (such as superoxide radical, hydrogen peroxide, and hypochlorite) will only exacerbate with chronic inflammation. Myeloperoxidase is another oxidizing agent linked to neurodegenerative and vascular diseases [58,59]. This has begun a trend in researching antioxidants as therapeutic remedies for these serious metabolic diseases [60].

Exercise can stimulate the acute inflammatory response as it is, in fact, damaging tissues on a cellular level [61]. As discussed previously, free radicals and reactive species are possible initiators and products of the inflammatory response. There are a number of interactions between the inflammatory products and reactive species/free radicals; stress hormones such as cortisol are products of the inflammatory response and further rely on redox balance for synthesis. The association between oxidative stress and the inflammatory response in exercise might further explain adaptation to exercise; the body responds to acute inflammation in such a way for the produced antigens to become less harmful [46]. This adaptation might rollover to explaining exercise adaptation, as well.

### 2.4. Exercise-Induced Oxidative Stress

Exercise can be very beneficial to health [62,63], but may also produce dangerous compounds. Davies was able to measure free radicals in isolated rat muscles via electron paramagnetic resonance following an endurance-exercise bout; exercise intensity increased over time resulted in an increase in free radicals [14]. Originally, researchers hypothesized that exercise could produce free radicals in one of two ways: the electron transport chain or ischemia-reperfusion. These two mechanisms are discussed below. There are now, however, other mechanisms of which are known to produce free radicals via exercise [64]. Included mechanisms are: the xanthine oxidase pathway, which produces the potent hydroxyl radical; the nicotinamide adenine dinucleotide phosphate oxidases pathway, which produces superoxide and hydrogen peroxide; and nitric oxide production, leading to other free radicals such as superoxide and peroxynitrite.

The electron transport chain is a biological mechanism that occurs in the mitochondria. As the name implies, electrons are transported down a gradient of complexes (complexes I through IV) via a series of redox reactions in order to generate the main energy source of adenosine triphosphate (ATP) in mammalian-biological systems. As mentioned earlier, oxygen is used as the final electron acceptor within this mechanism and can be sourced from respiration. Electrons can be “leaked” from this gradient leaving oxygen unpaired and oxidized into the reactive oxygen species, superoxide, or other free radicals [65]. Primary sites of free radical production within the electron transport chain are complexes I and III. During exercise, respiration can intensify as a direct response to the intensity of the activity. The increase in respiration results with an increase in systemic oxygen and electron transport chain activity, especially within the lungs [66,67]. This increase in systemic oxygen is thought to increase approximately 10–20 fold throughout the whole body [68] and 100–200 fold within the isolated, involving muscles [69]. Only about 0.15% of this oxygen, however, can be used to produce a reactive species [70].

**Figure 2:**
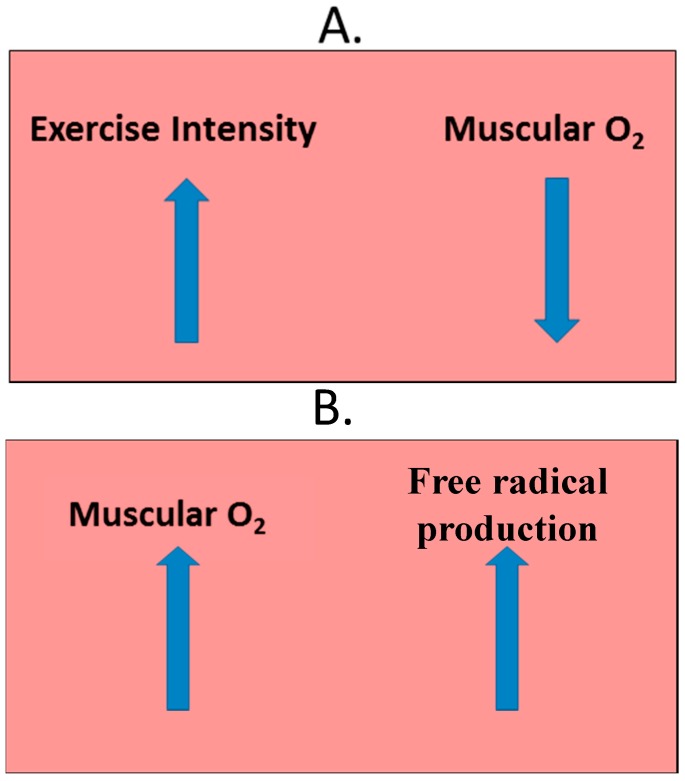
Skeletal muscle during (**A**) and after (**B**) exercise. (**A**) during exercise, as intensity reaches the point of VO_2max_ (maximum volume of oxygen uptake) muscular oxygen is depleted; (**B**) after exercise, muscular oxygen is repleted at such a fast pace potentially-damaging free radicals are produced [71,72].

Another process that can introduce free radicals is ischemia-reperfusion. When working muscles undergo exercise intensities that meet maximal concentrations of oxygen consumption (VO_2max_), their oxygen concentrations decrease. During the post-exercise period, these muscles will undergo a rapid increase in oxygen to make up for the lower concentrations present during the VO_2max_ period. At this time, reactive species are produced (Figure 2) [71,72]. If antioxidant defense systems are not capable of reducing such compounds to balance the redox-reaction’s equation, oxidative damage can occur.

While it may first appear that regular exercisers and athletes could be at risk from exercise-induced free radical influx, especially at intense levels and increased duration [73,74,75,76], other research suggests that regular exercisers and athletes adapt to such an influx over time. This adaptation would make them less susceptible to oxidative damage. Individuals who do not exercise regularly are more susceptible to oxidative damage but can become trained and develop a resistance to such damage [77]. The theorized mechanisms explaining why regular exercise improves adaption to oxidative stress include the upregulation of nuclear respiratory factor 1 (NRF-1) and of peroxisome proliferator-activated receptor gamma coactivator-1 alpha (PGC-1α). Both compounds depend on reactive species for activation of their signaling pathways that essentially lead to gene expression, phosphorylation, cell growth, and adaptations in the muscle potentially associated with improvements in exercise and sport performance [78,79,80,81,82]. Most research in exercise-induced oxidative stress has focused on aerobic exercise (e.g., running and bicycling), since oxygen is a major contributor to the synthesis of a reactive species. There is less research, however, regarding anaerobic exercise in the pool of exercise-induced oxidative stress.

### 2.5. Analyzing Exercise-Induced Oxidative Stress

One challenge in researching exercise-induced oxidative stress, and oxidative stress in general, is deciding which biomarker is most appropriate and if this biomarker is reflecting substantial oxidative damage to the organism. The different biomarkers reflecting lipid peroxidation, protein oxidation, and DNA oxidation and what they truly reflect should be considered. Malondialdehyde, for example, is a biomarker of lipid peroxidation, specifically polyunsaturated fatty acids; it does not necessarily reflect damage as accurately as F_2_-isoprostanes [2]. Another factor to consider when deciding the biomarker is the time of biospecimen collection; concentrations of certain biomarkers such as malondialdehyde will not peak immediately after the stressor [2]. It can also be difficult to choose which biospecimen (e.g., blood and saliva) to use for biomarker assessment. Blood is the most common for exercise-induced oxidative stress and biomarker extraction. Biomarkers present in blood include protein carbonyls [83,84,85,86]; total antioxidant capacity [87,88]; F_2_-isoprostanes [89,90]; malondialdehyde [91]; and Thiobarbituric Acid-Reactive Substances (TBARS), of malondialdehyde [92,93]. The presence of such blood biomarkers does not necessarily indicate damage is occurring in the surrounding cells/tissues; malondialdehyde, for instance, can be a product of actual damaged-tissue but also a product of reactive species. Different intensities of exercise and the exercise environment (e.g., high altitude) will also exhibit different concentrations of oxidative stress biomarkers [14,83]. Further research in these areas is warranted.

Saliva as a biospecimen has also been proposed for exercise-induced oxidative stress assessment [94,95]. Research in this area is limited and further research is warranted. Biomarkers used previously to examine oxidative stress and antioxidant status with saliva as a biospecimen include: total antioxidant capacity, uric acid, glutathione, advanced oxidation protein products, nitric oxide, and TBARS [96,97,98,99]; such biomarkers were used both in aerobic and anaerobic exercise conditions. Measuring and assessing oxidative stress in saliva has observed conflicting results using the TBARS assay, despite its popularity. The intensity of the exercise bout is the proposed culprit to this as the more intense exercise-bouts have observed significant increases in salivary TBARS [97] while less intense exercises have not [96]. The lack of sensitivity and specificity with the TBARS assay might also be cause for concern. Uric acid has been cited as a potential antioxidant and has consistently increased in saliva after exercise [96,98,99]; substantial increases might be due to the reduction of lipid peroxides. Free radicals, antioxidants, and all compounds in-between should continue to be studied to fully understand the balance in redox reactions and how it relates to health and exercise. Future research utilizing saliva as a biospecimen should have multiple, reliable biomarkers that are on both ends of the redox spectrum: for example, TBARS or malondialdehyde as oxidative-stress biomarkers and vitamin C and glutathione as antioxidant biomarkers.

## 3. Use of Salivary Biomarkers

### 3.1. The Basics of Saliva

Saliva is a fluid found in the oral cavity that has potential in identifying compounds under different diagnostic conditions in health, disease, and oxidative stress. It is comprised mostly of water [100] but also contains a variety of organic and inorganic components such as proteins, enzymes, electrolytes, immunoglobulins, hormones, and micronutrients such as vitamin C [101,102,103,104,105]. These components are essentially blood-based and permeate into the saliva due to different capillaries, acinar cells, and ductal cells [106]. Saliva is secreted into the oral cavity via major and minor glands with stimulation by the medulla [107]. The three major glands are the parotid gland, the sublingual gland, and the submandibular gland; the minor glands are the labial gland, the buccal gland, the lingual gland, and the palatal gland [108,109]. Each individual gland produces different amounts and flow rates of saliva with different compositions of its components at different times of the day. Such amounts and flow rates can also depend on if it is stimulated or unstimulated [103]. Some main functions of salivary components include the breakdown of local bacteria, digestion of carbohydrates and lipids, food lubrication, taste, and oral health [110,111,112].

An average individual can produce between 1 to 1.5 liters of saliva per day [113] making it a very appealing biospecimen in multiple sampling. Furthermore, saliva requires little to no safety-training for extraction and is a noninvasive alternative to blood and serum sample procedures. While it has been seen that some systemic biomarkers in health correlate well between saliva and blood in diagnosis (e.g., HIV [114]; hepatitis [115]; oral cancer [116]; periodontal disease [117,118]; and obesity [119]), saliva is not always the best biospecimen in that it does not always fully represent systemic concentrations such as with amylase, some proteomes, and phosphate [120]. 

### 3.2. Biomarkers of Oxidative Stress in Saliva

Technologies have advanced over the last century as well as the accuracy to identify salivary components. Biomarkers such as Malondialdehyde (MDA), 8-oxo-7, 8-oxo-2′-deoxyguanosine (8-oxo-dG), Thiobarbituric Acid-Reacting Substances (TBARS), total antioxidant capacity (TAC), uric acid, and protein carbonyls have been identified in the saliva due to such advancement [114,115,116,117,118,119]. Such biomarkers have been used in research studies focusing on oxidative stress and its association with exercise (discussed previously), health, and disease. Malondialdehyde has been used as a biomarker in recurrent aphthous ulceration (RAU) research with both serum and saliva used as a biospecimen [121,122]. Diabetes mellitus has also been studied with Malondialdehyde as a biomarker of oxidative stress in both serum and saliva despite lacking statistical significance in the control and experimental group concentrations [123]. These confounding results make it difficult to commit to saliva. Future research should focus on using the biomarkers/assays known to be accurate and reliable when assessing exercise-induced oxidative stress. TBARS, for example, is not necessarily the best to reflect oxidative stress or damage despite its ease in conducting. Free MDA and 9-oxo-dG might be considered more relevant as they are such with blood examination [2].

## 4. Vitamin C

### 4.1. Vitamin C as a Salivary Biomarker

Vitamin C, also known as ascorbic acid, is an essential micronutrient in that it is a necessity for certain species, including humans, to live. Scurvy (vitamin C deficiency) has been reported as early as 1550 B.C. [124] and led to vitamin C’s discovery in the early 1900s. It is also an antioxidant involved in a variety of hydroxylation reactions. Not long after its discovery was vitamin C found to be located in saliva. In 1935, vitamin C was measured in saliva at a 2.5 μg/mL concentration [125]. Other salivary vitamin C research studies, using salivary vitamin C as an index of vitamin status, helped in our understanding of vitamin C requirements [126,127,128,129,130,131,132]. Mäkilä and Kirveskari [126] are the researchers cited for the recorded range of salivary vitamin C concentration at 0.07–0.09 mg per 100 g of wet tissue; 0.07 mg came from the mixture of whole saliva while 0.09 mg came specifically from the parotid gland’s saliva. Saliva has been difficult to use as a biospecimen due to the failure in showing consistency of correlation with systemic biomarker concentrations. Vitamin C has fallen subject to this inconsistency, as well [128,133]. Research has repeatedly confirmed that other systemic biospecimens (e.g., blood, serum, and plasma) and vitamin C concentrations relate to dietary vitamin C intake [134]. When systemic concentrations of vitamin C fall to a deficient state, however, its salivary component is undetectable; this is likely a function of limited instrumental detection. Another success with saliva has come from the suggested relationship between vitamin C supplementation and salivary vitamin C [126]. Some diseases and disorders have also had significantly lower salivary vitamin C concentrations such as tuberculosis, parodontopathy, periodontitis, cancer, and leprosy [132,135,136,137,138,139]. There has been a lack of consistency in the assays used for determining salivary vitamin C status; the assays that have been used are derived from one of two methods: the dichlorophenolinodophenol method or the dinitophenylhydrazine method [140]. These methods utilize ascorbic acid’s antioxidant capabilities (that is to say that it can be reduced and oxidized easily) for colorimetry readings such as by a spectrophotometer. No study that looked at both exercise and salivary vitamin C was found.

### 4.2. Systemic Vitamin C

Vitamin C can be actively located in a variety of tissues at a variety of approximate concentrations [140]. Some examples of said tissues include the adrenal gland, plasma, and leukocytes. Plasma is often used as a biospecimen to evaluate systemic vitamin C status due to its response to the body’s systemic concentrations; however, leukocytes are the better tissue in regards to accurate measurements of bodily stores. Symptoms of vitamin C deficiency often occur when total body pools and plasma concentrations are 300 mg or less and 0.2 mg/dL or less, respectively [134,141]. Scurvy symptoms tend to arise when total body vitamin C pools are 300 mg or less [142,143]. Physiological changes related to scurvy can emerge when intakes are as little as 10 mg per day for a month. The Estimated Average Requirement (EAR) is set at 60 mg per day and 75 mg per day for females and males, respectively; this estimated requirement is set and recommended with the prevention of scurvy in mind. Roughly 37% of Americans are not taking in their EAR [144]. The suggested requirements are still debated upon and it is starting to become more theorized that problems might be developing due to such low intakes of vitamin C (and other essential nutrients). These problems include but are not limited to: harmful nitrosamine formation, low density lipoprotein oxidation, fatigue, and irritability [142]. Vitamin C intakes of 1.25 g (supplemented) per day fully saturate the blood plasma but roughly 50% of the vitamin is excreted in the urine [145]; this suggests that the more vitamin C taken in, the more that will be excreted. Around 200 mg is roughly the intake needed for full-body saturation with limited excretion. Doses larger than 500 mg will mostly be excreted [145]. Some of the foods that are higher in vitamin C include (contents of mg/100 g): peppers at 125–200 mg; kale at 120–180 mg; collard greens at 100–150 mg; broccoli at 90–150 mg; spinach at 50–90 mg; strawberries at 40–90 mg; cauliflower at 60–80 mg; and citrus fruits at 50 mg [146]. Scurvy as a vitamin C deficiency has not been completely eradicated despite its simplicity and known manifestations [147,148,149]. Some states that induce oxidative stress will also create lower plasma concentrations of the vitamin such as with smoking and diabetes [150,151,152,153,154,155]. These and similar findings have generated the discussion of other situations which require additional vitamin C from exogenous sources (e.g., food stuff and supplementation).

Vitamin C’s location in the body relates to its activity; as there are a variety of processes that require the vitamin to act as a cofactor, a co-substrate, and as an antioxidant in different tissues. It acts as a cofactor in the hydroxylation reactions that synthesize collagen, a protein found in a variety of tissues (skin, cartilage, ligaments, and tendons) necessary for structural and connective purposes. Proline and lysine residues utilize iron for the formation of 3-hydroxyproline, 4-hydroxyproline, and hydroxylysine [156,157,158]. Iron must be in its reduced state which is completed by vitamin C. Another hydroxylation reaction in which iron must be in its reduced state is the synthesis of carnitine, a compound required for fatty acid transportation into the mitochondria for energy utilization. The enzymes necessary for this reaction to occur are trimethyllysine hydroxylase and γ-butyrobetaine hydroxylase [159]; vitamin C is the agent which reduces iron back to its ferrous state for these reactions. The other metal that is often required in its reduced state during physiological processes is copper; vitamin C will reduce cupric ions to cuprous ions during norepinephrine synthesis. Norepinephrine is a catecholamine and neurotransmitter that has functions in an assortment of sympathetic and central nervous system processes. To exploit these processes, its hydroxylation involving vitamin C is required [160]. Vitamin C is also involved in tyrosine metabolism which requires both iron and copper in their reduced states for phenylalanine monooxygenase and 4-hydroxyphenylpyruvate hydroxylase, respectively, to be active as enzymes [161].

### 4.3. Vitamin C Supplementation for Exericse-Induced Oxidative Stress

Supplements in sports nutrition is an ever growing market in which vitamin C’s role still remains unknown. Research looking into vitamin C intake and exercise performance was conducted as early as the 1930s [162,163]. With vitamin C being an antioxidant and exercise being an inducer of oxidative stress, it is still one of the most popular supplements utilized and relevant for further research [164]. The evidence to suggest that vitamin C supplementation is beneficial, detrimental, or unavailing in exercise is unclear due to confounding results.

Systemic vitamin C will change as it reacts to different levels and types of oxidative stress, including exercise-induced oxidative stress [165,166,167,168]. Systemic vitamin C will quickly increase immediately after an exercise bout [166,167,168]. A negative feedback loop will occur within a couple of days after the exercise and results in a decrease in systemic concentrations [169,170,171]. The decrease in vitamin C might be its distribution and utilization within redox reactions of the affected tissues (e.g., reducing oxidative stress and/or recycling antioxidants) [172]. Daily training, as seen in athletes and avid exercisers, might then cause a continuous decrease in systemic vitamin C. This might generate a need for an increase in exogenous sources since low vitamin C is related to fatigue and decreased exercise performance. Whether the negative feedback loop normalizes or if specific exercise/sport intensities create different systemic vitamin C concentrations should be looked into further.

Vitamin C can be found in a variety of tissues that undergo exercise-induced oxidative stress. Exercise induced-oxidative stress then can potentially be reduced by the antioxidant, suggesting a use for its supplementation. The literature has conflicting views as far as this issue goes. Results to suggest vitamin C supplementation can attenuate oxidative biomarkers that are increased via exercise have been observed [173,174,175,176]. Whether these attenuations are necessary or even desired has yet to be determined since exercise adaptations might be reliant upon free radicals and reactive species at some sort of concentration. Reducing these compounds might then be to the contrary of the overall goal of exercise improvement. Studies have examined vitamin C supplementation alongside a variety of other antioxidants and their effects on oxidative stress and exercise adaptation on a cellular level (NRF-1 and PGC-1α) [177,178,179]. There is limited research examining these cellular pathways and vitamin C supplementation on its own in humans and should be examined further as it has been examined in rats [180]. There is reason to believe, however, that vitamin C will inhibit the NRF-1 and PGC-1α pathways, resulting in adaptation and improvement hindrance [180]. This would, again, negate the point of antioxidant supplementation within exercise and sport.

Exercise performance can be measured in a variety of different indices: VO_2max_, work capacity, distance for time, overall distance, muscle force, delayed fatigue, and muscle function to name a few. Supplementation of vitamin C, at doses from 500 mg to 1500 mg, has demonstrated improvements in indices of exercise performance; specifically in regards to timed exercise [181,182,183], delayed fatigue [184], and VO_2max_ [185]. There is research, however, that would suggest that vitamin C can compromise such performance indices, even at varying doses of 400 mg and 1000 mg [180,186]. Vitamin C supplementation without improvement or compromise in exercise performance has also been observed [187,188]. The continuance of its supplementation despite inconsistent results, even at similar doses, warrants future research on vitamin C in exercise. Markers of exercise performance have been lower in individuals with suboptimal vitamin C intakes; such individuals have also encountered higher concentrations of oxidative-stress biomarkers [189,190,191,192]. This is the only definitive situation for which supplementation of vitamin C has been suggested [193,194].

## 5. Future Research

A potential biospecimen that could be easy to obtain for measuring oxidative stress is saliva. It has been utilized to examine oxidative stress within aerobic and anaerobic exercise conditions; however, the only saliva biomarker consistently affected to reflect oxidative stress has been uric acid. Uric acid is an antioxidant that does not necessarily verify oxidative stress or oxidative damage; multiple salivary biomarkers for oxidative stress should be examined at one time. A significant increase in salivary TBARS was not observed but was so in plasma after resistance training, suggesting resistance training can induce oxidative stress [96]. Salivary TBARS might be more useful when higher intensity exercises are involved [97]. A more commonly recognized and accurate biomarker should be used in representing oxidative stress, such as malondialdehyde via HPLC. To fully depict the redox status during exercise while utilizing saliva as a biospecimen, an antioxidant biomarker should be measured alongside an oxidative-stress biomarker. One well-established antioxidant to be found in saliva is vitamin C. There has been little research conducted in order to suggest that systemic vitamin C can be represented by its salivary measurement. The amount of vitamin C taken in through the diet might or might not affect its salivary content but will increase its systemic content [129,195]. Vitamin C is one of the more popular supplements used in sports and exercise for a multitude of reasons, making it a relevant area for research still. Vitamin C supplementation has little to no evidence to suggest any benefit over the recommended estimated average requirement. Salivary vitamin C should be further researched in an exercised setting as it has not been fully examined.

An area of exercise that is lacking in oxidative stress and vitamin C research is resistance training. Resistance training is becoming more popularly performed in a variety of sport and exercise regimens; it does well at developing and indicating muscle strength and force. Oxidative stress has been induced via resistance exercise [96]. A popular combination in research now, and that has been used with resistance training, is supplementing vitamin C alongside other antioxidants to reduce oxidative stress and increase exercise performance [196,197,198]. Vitamin C alone has had limited research exposure in this area and should be warranted to do so. A positive result was observed with a high dose of vitamin C supplementation in eccentric exercise (a form of resistance training) [199]; a substantial reduction in muscle soreness was observed.

Possible areas of more research in resistance training might be looking at the roles of calcium, H_2_O_2_, and vitamin C’s role in such involved processes. Calcium is an important mineral in the body with tasks involving bone health and muscle contraction and force. Reactive oxygen species might have an effect on muscle force on a cellular level [200,201]. Vitamin C is involved in calcium absorption, therefore possibly having a secondary influence on the cellular system of muscle force. H_2_O_2_ and other reactive species might also inhibit muscular force [202]. Vitamin C is one of the more common antioxidants to reduce H_2_O_2_. The research on vitamin C hindering, enhancing, or having no effect on muscular force is limited and needs further investigation. Whether certain doses of vitamin C intake significantly affect sport and exercise performance should also be taken into consideration as some sort of hormesis effect might be at play.

**Figure 3:**
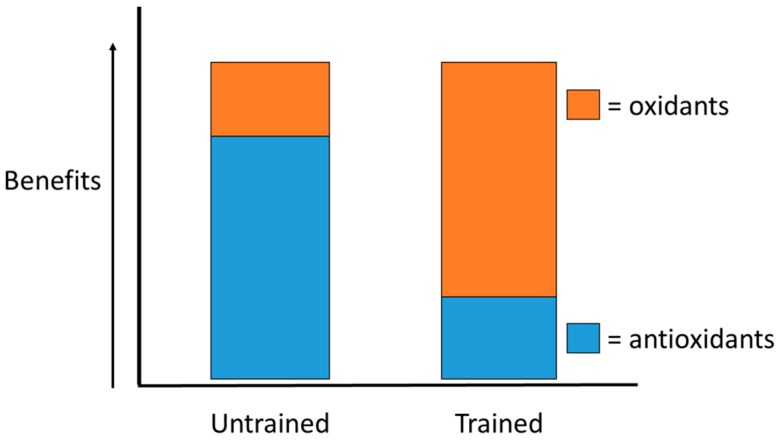
Balance between antioxidants and oxidants in untrained and trained individuals in as it relates to exercise benefits. An individual who is less experienced in exercise might find more of a benefit with higher concentrations of systemic antioxidants (such as via supplementation); an individual who is more trained in exercise might find more of a benefit with higher concentrations of systemic oxidants (free radicals/reactive species with their respective roles in exercise adaptation [203].

Oxidative stress is a very normal and sensitive homeostatic event which can be damaging overall. Whether oxidative stress is the primary cause of this damage or just a secondary event is left to be researched further in a variety of different settings which includes exercise. In exercise, it is possible that biomarkers which reflect oxidative stress might play a part in important sport and exercise adaptations on a cellular and molecular level. Future researchers should examine whether such biomarkers reflect real damage or are necessary for exercise adaptation. Certain circumstances might include untrained persons wanting to start exercising or competing in sport. An untrained person might experience oxidative stress as a somewhat damaging situation at first; adaptations that occur over time might result in a beneficial outcome on a cellular and physiological level (Figure 3). A perfect balance between oxidative stress induced via exercise and available antioxidants has been proposed [203]. Whether antioxidant supplementation would alleviate oxidative stress in untrained persons, beneficially, should be further examined. Such benefits might include quicker adaptation to the stress or a reduction in the resulting damage. There might be a certain concentration in which oxidative stress mediators are beneficial before they become harmful; a set parameter suggesting normal concentrations might alleviate this concern. A variety of different variables can affect exercise adaptation and general performance over time (e.g., diet, training intensity, training schedule, level of skill, and performance supplementation); these variables should be controlled in future research.

## 6. Conclusions

Saliva is a potential biospecimen to be used in a variety of research settings including oxidative stress. It has multiple benefits as a biospecimen which include its easy obtainability, its little required training needed for collection, the body’s ability to produce its unlimited quantity, and its possible reflection of some systemic biomarkers that might identify health status. There has been research to suggest that it does not reflect some biomarkers that are more commonly used in identifying health status. The research regarding exercise and oxidative stress with salivary biomarkers is heavily lacking. Resistance training is lacking more so in research regarding oxidative stress, salivary biomarkers, and vitamin C supplementation. The evidence in recommending supplementation of vitamin C is confounding. The only positive results in vitamin C supplementation are with individuals who have low vitamin C intakes. Ironically, 37% of the population isn’t consuming the estimated average vitamin C requirement; this could theoretically cause symptoms such as decreased exercise-performance and fatigue. Such a requirement can be satisfied by consuming the recommended servings of 5 fruits and vegetables every day. There is evidence to suggest that this estimated requirement should be increased to 95 mg and 200 mg per day for females and males, respectively [134,142].
